# Internet Search Results for Older Adult Physical Activity Guidelines: Scoping Review

**DOI:** 10.2196/29153

**Published:** 2022-01-13

**Authors:** Samantha M Harden, Anna Murphy, Kathryn Ratliff, Laura E Balis

**Affiliations:** 1 Physical Activity Research and Community Implementation Laboratory Department of Human Nutrition, Foods, and Exercise Virginia Tech Blacksburg, VA United States; 2 Physical Activity Research and Community Implementation Laboratory Virginia Tech Blacksburg, VA United States; 3 Balis Consulting Group LLC Little Rock, AR United States

**Keywords:** dissemination, information seeking, health communication, elderly

## Abstract

**Background:**

Older adults seek health-related information through casual internet searches. Yet, researchers focus on peer-reviewed journals and conference presentations as primary dissemination strategies. Representatives of mass media are alerted (passive diffusion) of new studies or recommendations, but the veracity of the information shared is not often analyzed, and when it is, the analysis is often not comprehensive. However, most older adults do not have access to peer-reviewed journal articles or paid subscription services for more reputable media outlets.

**Objective:**

We aimed to determine what information was readily available (ie, open access) to older adults who may casually search the internet for physical activity recommendations.

**Methods:**

We performed a 6-part scoping review to determine the research question and available evidence, and extract data within open-access top hits using popular online search engines. Results were categorized by a dissemination model that has categories of sources, channels, audience, and messages.

**Results:**

After the iterative search process, 92 unique articles were included and coded. Only 5 (5%) cited physical activity guidelines, and most were coded as promoting healthy aging (82/92, 89%) and positive framing (84/92, 91%). Most articles were posed as educational, but the authors’ credentials were rarely reported (ie, 22% of the time). Muscle strengthening and balance components of the physical activity guidelines for older adults were rarely reported (72/92, 78% and 80/92, 87%, respectively) or inaccurately reported (3/92, 3% and 3/92, 3%, respectively).

**Conclusions:**

Inconsistent messages lead to mistrust of science and public health representatives. This work highlights the lack of evidence within existing open-access resources. Further efforts are needed to ensure evidence-based public health messages are in the sources and channels older adults are using to inform their knowledge and behaviors.

## Introduction

The strategic spread of evidence-based information is recognized as a necessity, replacing the passive diffusion of information [1-6]. This active knowledge exchange may reduce unnecessary duplication, increase the reach to those most in need of intervention, and improve knowledge and health equity [7,8]. In a time of fake news, social media influencers, and mistrust of scientific evidence, what is disseminated to specific audiences and how is it disseminated have become vital lines of scientific inquiry [9,10]. Thus, dissemination research investigates how, when, by whom, and under what circumstances research evidence is spread among agencies, organizations, and frontline workers who provide public health and clinical services [1,11,12].

A key challenge of dissemination is the discrepancy between how researchers disseminate findings (academic journals and academic conferences) and how end users (community members) seek information. For example, even if an individual seeks evidence-based information, a peer-reviewed journal article may not be open access. While many institutions have access agreements with journals, the public is not generally granted access. Furthermore, even if access is more “open,” people are not typically seeking health-related information through peer-reviewed journal articles.

For example, older adults, a priority population for health promotion efforts, seek information about health through both living and nonliving sources [13]. They report greater trust in living sources (eg, clinicians or friends) due to the ability to actively discuss their health. However, when living sources are unavailable, many older adults report relying on general internet searches and have expressed concerns about their ability to access the veracity of information [14]. Many investigations have explored “getting the word out” [4] and “getting the message across” [15], as well as the information seeking practices of end users [13,14]. However, less attention has been paid to what is actually available after these casual internet searches and the degree to which the information found is evidence-based.

One health behavior older adults seek information on is physical activity. Physical activity compliance decreases the risk for chronic conditions (including obesity, hyperglycemia, hypercholesterolemia, and hypertension) [16]. There are specific multifaceted guidelines for older adults (those aged ≥65 years) within the Physical Activity Guidelines for Americans (PAGA) [17]. Balance, flexibility, motor coordination, strength training, and cardiovascular components [18] typically deteriorate as we age [19,20]. Therefore, the PAGA for older adults include 150 minutes of moderate intensity aerobic activity, 2 days of muscle strengthening, and balance activities [17]. Yet, 79% of older adults are not meeting the guidelines for aerobic activity, strength, and balance [21-23]. This demonstrates a research-to-practice gap and a need for improved dissemination efforts.

The objective of this work was to understand the existing PAGA messages that older adults receive and how those messages may be tailored to better reach older adults and, ultimately, inform physical activity behaviors. This paper shares the iterative scoping review process for identifying where and what information older adults may be receiving related to the PAGA.

## Methods

### Overview

A modified version of the staged approach of a scoping review was employed. It involved the following: (1) identifying the research question, (2) consultation, (3) identifying relevant studies, (4) study selection, (5) charting the data, and (6) collating, summarizing, and reporting the results [24]. In a traditional scoping review, consultation is the final step in the analysis. Rather than asking older adults and older adult PAGA researchers at the end of the search process, we used their feedback to inform the search process. The review protocol is available upon request. The PRISMA-ScR (Preferred Reporting Items for Systematic Reviews and Meta-Analyses extension for Scoping Reviews) checklist is provided in Multimedia Appendix 1.

### Step 1: Identifying the Research Question

The first observation was that while most Americans do not meet physical activity guidelines, few Americans meet strength training recommendations when compared with aerobic activity guidelines. One hypothesis was that strength training recommendations are less frequently reported in mass media. However, before understanding what has been reported in mass media, honing in on the appropriate outlets was necessary. Older adults search for information through “simple surfing” on the internet [25], but the web browser, search engine, and search terms have not been reported in the literature. Therefore, the final research question is as follows: If older adults engage in simple surfing, what information would they receive about the PAGA?

### Step 2: Consultation

We distributed a Qualtrics survey through the Virginia Tech Older Adult Research Registry (N= 163). There were 17 bounce-back emails and 32 (22%) survey respondents. The participants were 66 to 85 years of age (mean 73 years, SD 5.01 years). When asked how they would search the internet for information on exercise, the responses included questions about proper form, improved balance and strengthening, safe exercises for the older adult age range, exercises to target specific parts of the body, and exercises to prevent or improve physical conditions. Most (21/32, 66%) of the participants reported using Google Chrome as their web browser, but some also used Internet Explorer (8/32, 25%), Firefox (4/32, 12.5%), Safari (4/32, 12.5%), or Microsoft Edge (3/32, 9%). Google Search was used by 100% of the participants, with only a few also using Bing (3/32, 9%) and Yahoo (3/32, 9%).

### Step 3: Identifying Relevant Materials

First, we selected our search terms based on previous physical activity reviews [26-29] and terms older adults prefer when searching for information specific to their age group [30]. Our final search terms were as follows: Physical Activity OR exercise OR movement OR physical activity guidelines OR activities OR fitness and (Older Adults OR seniors OR senior citizen OR elderly OR retiree OR 65 OR geriatric*).

Second, we selected our web browser. The research team tested the search terms on 3 different computers, at different IP addresses, and found that the searches within each search engine did not differ based on the browser (Google Chrome, Firefox, and Safari). Therefore, only the web browser Mozilla Firefox was used for the final search protocol.

Third, we selected the most relevant search engines based on the respondents from the Older Adult Research Registry and the extant data (Google Search, Bing, Yahoo, and Duck Duck Go). In general, 71% of internet searches are conducted through Google Search, and 68% of searchers click on results within the first 5 listings of the first page. This rate drops to only 4% of searchers viewing pages 6 to 10 [31]. Thus, to be overly conservative, the first 10 articles per search engine were extracted. The search included anything from January 1, 2008, to January 31, 2019. All articles had to be open access (ie, no payment or subscription for viewing).

### Step 4: Material Selection

Articles were eligible for review if the content included recommendations on physical activity or exercise for older adults and was open access (free to view). The initial search resulted in 583 articles. Duplicates were removed (n=153). As many of the articles were not scientific (ie, peer reviewed or structured with an abstract), the typical abstract review process of a systematic review was replaced with a title review and then (1) a visual assessment of the landing page, which eliminated many articles (eg, advertisements, dumbbell purchase, and dead links) and (2) a review of the text (for key terms such as physical activity or older adults). If an article did not meet the eligibility criteria (eg, not about older adults or exercise), it was excluded (n=43). Full-text review was conducted on 110 articles, with 92 meeting the final eligibility criteria. Each article was independently coded. Half of the articles (n=45) were coded by 2 investigators to establish interrater reliability. Once interrater reliability was over 85%, an additional 10% of the articles were coded to ensure strong interrater reliability. The remaining articles were coded by 1 author (AM).

## Results

### Step 5: Charting the Data

The data extraction form was built around the categories of “getting the word out” [4,32] as follows: (1) *source* can be operationalized as who is sharing the message (ie, credential); (2) *message* is the “what” (content); (3) *audience* is to whom the message is intended (eg, characteristics and values); and (4) *channel* is where the information is provided (eg, the location of the content). In addition, items were crafted in alignment with the Agency for Healthcare Research and Quality guidance for “Communication and Dissemination Strategies To Facilitate the Use of Health-Related Evidence” [33]. For example, 1 variable was the purpose, and independent coders established whether it was educational, entertainment, commercial, or other. Variables also included antiaging and healthy aging, with the former attempting to prevent the effects of aging, and the latter embracing one’s age and the effects. Framing was divided into positive or negative categories. Positive focused on the benefits of exercise training, while negative highlighted the unfavorable effects of not partaking in exercise. Audience, another variable, included tailoring for age (older adult specific or not), sex (male or female), culture (ie, social behavior or customs), and attitudes, norms, or beliefs. The complete list of items is available upon request, and a summary of the data is presented in Table 1. The quality of evidence is summarized in Table 2.

**Table 1:** Summary of dissemination source, channel, message, and audience.

Variable					Value (N=92), n (%)
**Channel**					
	**Type**				
		Internet article	71 (77)		
		Blog	6 (7)		
		Commercial	1 (1)		
		Journal	14 (15)		
	**Resources**				
		Community	4 (4)		
		Commercial	10 (11)		
		Government	1 (1)		
		Peer review	1 (1)		
		Not reported	76 (83)		
	**Number of cite visitors/reader (reach)**				
		Not reported	90 (98)		
**Source**					
	**Author credentials**				
		Not reported	72 (78)		
		PhD or academic	3 (3)		
		Medical doctor (MD, DO)	4 (4)		
		Physical therapist	1 (1)		
		Personal trainer	5 (5)		
		Freelancer	3 (3)		
		Aging/health expert	1 (1)		
		Other	3 (3)		
	**Quotes**				
		Clinician	5 (5)		
		Researcher	6 (7)		
		Instructor	6 (7)		
		Older adult	1 (1)		
		No quote	74 (81)		
**Message**					
	**Purpose**				
		Commercial	4 (4)		
		Education	83 (90)		
		Entertainment	1 (1)		
		N/A^a^	1 (1)		
		Other	3 (3)		
	Cite PAGA^b^ 2008, Yes			5 (5)	
	Cite PAGA 2018, Yes			5 (5)	
	**Include aerobic requirements**				
		Yes	8 (9)		
		Yes, but inaccurately	5 (5)		
		No	72 (78)		
		Other^c^	7 (8)		
	**Include strength requirements**				
		Yes	11 (12)		
		Yes, but inaccurately	3 (3)		
		No	72 (78)		
		Other^c^	6 (7)		
	**Include balance requirements**				
		Yes	7 (8)		
		Yes, but inaccurately	3 (3)		
		No	80 (87)		
		Other^c^	2 (2)		
	**Aging**				
		Antiaging	5 (5)		
		Healthy aging	82 (89)		
		N/A	5 (5)		
	**Framing**				
		Negative	6 (7)		
		Positive	84 (91)		
		Undiscernible	2 (2)		
**Audience**					
	**Tailoring, n(%)**				
		Age	47 (51)		
		Sex	2 (2)		
		Culture	2 (2)		
		Attitudes, norms, and beliefs	83 (90)		
	Narrative shared (eg, testimonial, experience, and hypothetical or actual story)			5 (5)	

^a^N/A: not applicable.

^b^PAGA: Physical Activity Guidelines for Americans.

^c^Other indicates recommendations that are scientific but not the Physical Activity Guidelines for Americans (eg, American College of Sports Medicine).

**Table 2:** Quality of evidence.

Variable			Value (N=92), n (%)
**Strength of evidence**			
	High	25 (27)	
	Medium	24 (26)	
	Low	43 (47)	
**Risk of bias**			
	High	37 (40)	
	Medium	29 (32)	
	Low	26 (28)	
**Consistency**			
	High	28 (30)	
	Medium	40 (44)	
	Low	24 (26)	
**Precision**			
	High	22 (24)	
	Medium	31 (34)	
	Low	39 (42)	
**Directness**			
	High	24 (26)	
	Medium	40 (44)	
	Low	28 (30)	
**Net benefit**			
	High	22 (24)	
	Medium	32 (35)	
	Low	38 (41)	
**Applicability**			
	High	11 (12)	
	Medium	57 (62)	
	Low	24 (26)	

### Step 6: Collating, Summarizing, and Reporting the Results

**Figure 1:**
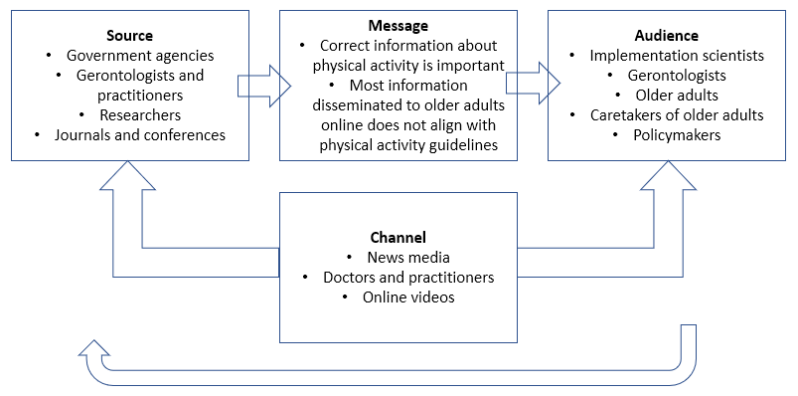
Dissemination source, channel, message, and audience for the Physical Activity Guidelines for Americans.

In total, 92 unique articles were included. Example titles were “Exercise for Older Adults,” “Over 65? Cardio Exercise or Weight Training?” and “The Basics of Training Older Adults.” Thirty-three articles did not include their publication date, but for those that did, they ranged from 1999 to 2020, with the highest proportion being from the year after the PAGA 2018 (2019; 11%). Articles took 10.97 (SD 5.2) minutes to read. The full summary of article features across the dissemination categories of source, channel, audience, and message can be found in Table 1. Only 5 (5%) of the articles reported PAGA editions (2008 or 2018). A majority of the articles were coded as promoting healthy aging (82/92, 89%) and positive framing (84/92, 91%). Most articles were posed as educational, but the authors’ credentials were rarely reported. The specific components of the PAGA for older adults for aerobic activity, muscle strengthening, and balance were usually not reported (72/92, 78%; 72/92, 78%; and 80/92, 87%, respectively) or inaccurately reported (5/92, 5%; 3/92, 3%; and 3/92, 3%, respectively). Figure 1 provides a summary of the source, message, audience, and channel to increase PAGA dissemination to older adults.

## Discussion

This work aimed to understand messages older adults may receive when seeking information related to being physically active. Using a modified scoping review methodology, these internet search results do not represent traditional articles; however, they represent open-access information end users are often receiving to inform their decision-making. Rather than reporting by article, we report as an aggregate the general messages sent to older adults regarding physical activity recommendations. The significant contribution of this work is that, in alignment with a recent review of initial mass media coverage of the PAGA [34], the information available to the public was incomplete and often inaccurate. Notably, while search engines, terms, and top hits have some variability, this work was conducted years after the release of the second edition of the PAGA, so the articles consisted of evergreen content of ongoing relevance [35].

The data were extracted based on the source, message, audience, and channel of dissemination [4]. Most notably, this work found that many common searches resulted in articles that were not evidence-based or evidence-informed [36]. This bold statement is based on the fact that approximately 80% of the articles did not cite any edition of the PAGA or author credentials. This is concerning for 2 primary reasons. First, it is unsurprising that most older adults (90%) are not meeting the PAGA since awareness is essential to any transformation of human behavior [37]. The study presented here unearthed that multicomponent exercise recommendations were rarely disseminated through popular search engines and terms. Second, the sources of these data do not quote experts in the field or share their own credentials. It is important to note that we are not claiming that the authors of the articles do not have the credentials necessarily, just that the credentials were not shared with the general audience. The link between popular sources and channels and the evidence-base is necessary, or the general public may continue to be misinformed about health-enhancing physical activity (eg, type, intensity, and duration).

This issue is not isolated to the PAGA and the United States. Physical inactivity is a growing global problem [38]. The World Health Organization’s Global Strategy on Diet, Physical Activity, and Health provided guidance to member states in establishing national recommendations and plans [39], resulting in the adoption of guidelines across the globe [40-42]. However, it is unknown if the resultant guidelines have been successfully disseminated and implemented. For example, older adults in Ghana reported that they were unaware of the guidelines and how to meet them [43]. More work is needed to determine appropriate communication channels (eg, websites, publications, and mass media) and messages for disseminating the guidelines to both public health practitioners and members of the public [44].

Another challenge in disseminating evidence-based public health information is competing for search engine rankings with those who write blog posts or articles for marketing rather than educational purposes. While many of the articles identified in this review were classified as educational rather than commercial (ie, they did not include links or product advertisements), the sources were for-profit companies rather than nonprofits or educational institutions. Thus, the education is provided for the purpose of drawing in website traffic to increase sales. This distinction is important as those who write for commercial websites may be better trained in search engine optimization (SEO) than those who write for purely educational websites [45].

SEO refers to the methods and techniques used to improve search rankings and increase website traffic [46,47]. One strategy involves selecting specific highly searched terms or phrases and incorporating them throughout an article with the goal of making the article more search engine friendly [45]. For example, “benefits of exercise” has a higher search volume than “benefits of physical activity” [48]. To improve search rankings, “benefits of exercise” would be used in the title and anchor text (words that are hyperlinked), and multiple times throughout the body of the article.

As search engine users typically only view the first page results [49], articles written without using SEO strategies may appear beyond the scope of what most audiences read and may never be disseminated to the public. To combat this, public health practitioners and researchers can improve dissemination efforts by learning to use SEO [45,49,50]. However, it is recognized that replacing important terms (eg, changing “physical activity” to “exercise”) compromises the scientific integrity of the writing. The best option may be to strive for a balance of using highly searched keywords and phrases when appropriate while also maintaining scientific accuracy.

In addition to the channel and source, the type of message is relevant for behavior change. For example, whether the message is framed positively or negatively influences information retention and behavior change [15,51]. Furthermore, positive messages are more influential for older adults compared to younger adults [15]. Our initial search included articles from *The New York Times* that used negative framing (eg, “25 Again? How Exercise May Fight Aging”). As *The New York Times* requires a fee for access (after 1-3 free article views), the articles were excluded. Using the open-access review criteria, there was a surprising shift toward a goal and theme of healthy aging. After concluding the review, articles with a healthy aging message (82/92, 89%) far surpassed antiaging articles (5/92, 5.4%). We saw the same trend with negative (6.5%) versus positive framing (91.3%) of the articles. Negative framing often focused on the poor outcomes from a lack of exercise and the possible downward spiral in old age. Positive framing concentrated on the benefits and additions that physical activity can provide to older adults. With regard to tailoring, the a priori tailoring categories consisted of age, sex, and culture, as well as norms, attitudes, and beliefs. There was no specific variable for tailoring for individuals with lower health literacy, which is a limitation of this work.

One further limitation of this review is the inability to be replicated due to the unique nature of the searches. If this review is conducted again, it would likely produce different top hits on the search engines. This review also identified a low response rate (22%) from the Older Adult Research Registry during our consultation process. It is likely that we received interest from the most digitally affluent older adults. The responses could have also been from older adults who were particularly interested in the topic of physical activity research or those who were more knowledgeable about internet searches. This, coupled with the lack of tailoring for older adults with low literacy, may reduce the applicability of the findings and further contribute to disparities of older adults meeting the PAGA [52,53]. This review focused on the dissemination of information about physical activity to older adults through the internet, but there is room for more work to be done in other forms of communication (eg, books, newspapers, and television). One particular challenge of this work was translating typical peer-review journal article critiques and methodologies to grey literature. For example, the risk of bias assessment was particularly challenging. The research team developed a coding guide to clarify operationalization of each of these constructs within this context. Therefore, these results should be interpreted with caution. This is particularly notable for the articles that were not intended to be evidence-based. Finally, while this work focused on the dissemination of PAGA, further work may be warranted to determine the degree to which the American College of Sports Medicine’s Exercise is Medicine initiative is included in more colloquial articles. That said, this study identified that low-quality information is being shared with the public and is being potentially trusted as accurate.

## Appendix

Multimedia Appendix 1 Preferred Reporting Items for Systematic Reviews and Meta-Analyses extension for Scoping Reviews (PRISMA-ScR) Checklist.

## Abbreviations

PAGA: Physical Activity Guidelines for Americans
SEO: search engine optimization

## References

[1] Rabin BA, Brownson RC, Haire-Joshu D, Kreuter MW, Weaver NL (2008). A glossary for dissemination and implementation research in health. J Public Health Manag Pract.

[2] Birken SA, Bunger AC, Powell BJ, Turner K, Clary AS, Klaman SL, Yu Y, Whitaker DJ, Self SR, Rostad WL, Chatham JRS, Kirk MA, Shea CM, Haines E, Weiner BJ (2017). Organizational theory for dissemination and implementation research. Implement Sci.

[3] Glasgow RE, Vinson C, Chambers D, Khoury MJ, Kaplan RM, Hunter C (2012). National Institutes of Health approaches to dissemination and implementation science: current and future directions. Am J Public Health.

[4] Brownson RC, Eyler AA, Harris JK, Moore JB, Tabak RG (2018). Getting the Word Out: New Approaches for Disseminating Public Health Science. Journal of Public Health Management and Practice.

[5] Estabrooks PA, Brownson RC, Pronk NP (2018). Dissemination and Implementation Science for Public Health Professionals: An Overview and Call to Action. Prev Chronic Dis.

[6] Thomas JD, Flay BR, Cardinal BJ (2018). Are Physical Activity Resources Understandable as Disseminated? A Meta-Analysis of Readability Studies. Quest.

[7] Strayer T, Balis L, Harden S (2020). Partnering for Successful Dissemination: How to Improve Public Health With the National Cooperative Extension System. Journal of Public Health Management and Practice.

[8] Norton WE, Chambers DA (2020). Unpacking the complexities of de-implementing inappropriate health interventions. Implement Sci.

[9] Brownson RC, Burke TA, Colditz GA, Samet JM (2020). Reimagining Public Health in the Aftermath of a Pandemic. Am J Public Health.

[10] Swire-Thompson B, Lazer D (2020). Public Health and Online Misinformation: Challenges and Recommendations. Annu Rev Public Health.

[11] Brownson R, Colditz G, Proctor E (2012). Dissemination and Implementation Research in Health: Translating Science to Practice.

[12] Burke JG, Lich KH, Neal JW, Meissner HI, Yonas M, Mabry PL (2015). Enhancing dissemination and implementation research using systems science methods. Int J Behav Med.

[13] Turner AM, Osterhage KP, Taylor JO, Hartzler AL, Demiris G (2018). A Closer Look at Health Information Seeking by Older Adults and Involved Family and Friends: Design Considerations for Health Information Technologies. AMIA Annu Symp Proc.

[14] Chaudhuri S, Le T, White C, Thompson H, Demiris G (2013). Examining health information-seeking behaviors of older adults. Comput Inform Nurs.

[15] Shamaskin AM, Mikels JA, Reed AE (2010). Getting the message across: age differences in the positive and negative framing of health care messages. Psychol Aging.

[16] Sierra F, Hadley E, Suzman R, Hodes R (2009). Prospects for life span extension. Annu Rev Med.

[17] Piercy KL, Troiano RP, Ballard RM, Carlson SA, Fulton JE, Galuska DA, George SM, Olson RD (2018). The Physical Activity Guidelines for Americans. JAMA.

[18] Kahn E, Ramsey L, Brownson R, Heath G, Howze E, Powell K, Stone E, Rajab M, Corso P (2002). The effectiveness of interventions to increase physical activity. A systematic review. Am J Prev Med.

[19] Lord SR, Castell S (1994). Physical activity program for older persons: Effect on balance, strength, neuromuscular control, and reaction time. Archives of Physical Medicine and Rehabilitation.

[20] Perry BC (1982). Falls among the elderly: a review of the methods and conclusions of epidemiologic studies. J Am Geriatr Soc.

[21] Ashe MC, Miller WC, Eng JJ, Noreau L, Physical ActivityChronic Conditions Research Team (2009). Older adults, chronic disease and leisure-time physical activity. Gerontology.

[22] Sun F, Norman IJ, While AE (2013). Physical activity in older people: a systematic review. BMC Public Health.

[23] Jefferis BJ, Sartini C, Lee I, Choi M, Amuzu A, Gutierrez C, Casas JP, Ash S, Lennnon LT, Wannamethee SG, Whincup PH (2014). Adherence to physical activity guidelines in older adults, using objectively measured physical activity in a population-based study. BMC Public Health.

[24] Levac D, Colquhoun H, O'Brien KK (2010). Scoping studies: advancing the methodology. Implement Sci.

[25] Lucas B (2007). 'Simple surfing' where people can get information on health. Nurs Older People.

[26] Galaviz KI, Harden SM, Smith E, Blackman KC, Berrey LM, Mama SK, Almeida FA, Lee RE, Estabrooks PA (2014). Physical activity promotion in Latin American populations: a systematic review on issues of internal and external validity. Int J Behav Nutr Phys Act.

[27] Burke S, Carron A, Eys M, Ntoumanis N, Estabrooks P (2006). Group versus individual approach? A meta-analysis of the effectiveness of interventions to promote physical activity. Journal of Sport & Exercise Psychology.

[28] Bhuiyan N, Singh P, Harden SM, Mama SK (2019). Rural physical activity interventions in the United States: a systematic review and RE-AIM evaluation. Int J Behav Nutr Phys Act.

[29] Harden SM, McEwan D, Sylvester BD, Kaulius M, Ruissen G, Burke SM, Estabrooks PA, Beauchamp MR (2015). Understanding for whom, under what conditions, and how group-based physical activity interventions are successful: a realist review. BMC Public Health.

[30] Chafetz PK, Holmes H, Lande K, Childress E, Glazer HR (1998). Older adults and the news media: utilization, opinions, and preferred reference terms. Gerontologist.

[31] Google Organic Click-Through Rates in 2014. MOZ.

[32] Strayer TE, Kennedy LE, Balis LE, Ramalingam NS, Wilson ML, Harden SM (2020). Cooperative Extension Gets Moving, but How? Exploration of Extension Health Educators' Sources and Channels for Information-Seeking Practices. Am J Health Promot.

[33] Communication and Dissemination Strategies To Facilitate the Use of Health-Related Evidence. Effective Health Care (EHC) Program.

[34] Maddock JE, Kellstedt D (2020). Initial mass media coverage of the 2nd edition of the physical activity guidelines for Americans. Prev Med Rep.

[35] Evergreen Content. Search Engine Journal.

[36] Sarkies MN, Bowles K, Skinner EH, Haas R, Lane H, Haines TP (2017). The effectiveness of research implementation strategies for promoting evidence-informed policy and management decisions in healthcare: a systematic review. Implement Sci.

[37] Ballew P, Brownson RC, Haire-Joshu D, Heath GW, Kreuter MW (2010). Dissemination of effective physical activity interventions: are we applying the evidence?. Health Educ Res.

[38] Kohl HW, Craig CL, Lambert EV, Inoue S, Alkandari JR, Leetongin G, Kahlmeier S (2012). The pandemic of physical inactivity: global action for public health. The Lancet.

[39] (2007). A guide for population-based approaches to increasing levels of physical activity : implementation of the WHO global strategy on diet, physical activity and health. World Health Organization.

[40] Kahlmeier S, Wijnhoven TMA, Alpiger P, Schweizer C, Breda J, Martin BW (2015). National physical activity recommendations: systematic overview and analysis of the situation in European countries. BMC Public Health.

[41] Holdsworth M, El Ati J, Bour A, Kameli Y, Derouiche A, Millstone E, Delpeuch F (2013). Developing national obesity policy in middle-income countries: a case study from North Africa. Health Policy Plan.

[42] Dietary and Physical Activity Guidelines for Ghana. PDF4PRO.

[43] Balis LE, Sowatey G, Ansong-Gyimah K, Ofori E, Harden SM (2019). Older Ghanaian adults' perceptions of physical activity: an exploratory, mixed methods study. BMC Geriatr.

[44] Balis L (2020). Stakeholder Input to Inform the Adaptation and Dissemination of Ghana’s Physical Activity Guidelines.

[45] Hanson C, Thackeray R, Barnes M, Neiger B, McIntyre E (2008). Integrating Web 2.0 in Health Education Preparation and Practice. American Journal of Health Education.

[46] Grappone J, Couzin G (2011). Search Engine Optimization (SEO): An Hour a Day.

[47] Ledford JL (2007). SEO: Search Engine Optimization Bible.

[48] Wordtracker.

[49] Modave F, Shokar NK, Peñaranda E, Nguyen N (2014). Analysis of the Accuracy of Weight Loss Information Search Engine Results on the Internet. Am J Public Health.

[50] Dunne S, Cummins NM, Hannigan A, Shannon B, Dunne C, Cullen W (2013). A method for the design and development of medical or health care information websites to optimize search engine results page rankings on Google. J Med Internet Res.

[51] Rosenblatt DH, Bode S, Dixon H, Murawski C, Summerell P, Ng A, Wakefield M (2018). Health warnings promote healthier dietary decision making: Effects of positive versus negative message framing and graphic versus text-based warnings. Appetite.

[52] Cohen SA, Greaney ML, Sabik NJ (2018). Assessment of dietary patterns, physical activity and obesity from a national survey: Rural-urban health disparities in older adults. PLoS One.

[53] Wolf MS, Gazmararian JA, Baker DW (2005). Health literacy and functional health status among older adults. Arch Intern Med.

